# Trends in Detection of Invasive Cancer and Ductal Carcinoma In Situ at Biennial Screening Mammography in Spain: A Retrospective Cohort Study

**DOI:** 10.1371/journal.pone.0083121

**Published:** 2013-12-23

**Authors:** Marta Román, Montse Rué, Maria Sala, Nieves Ascunce, Marisa Baré, Araceli Baroja, Mariola De la Vega, Jaume Galcerán, Carmen Natal, Dolores Salas, Mercedes Sánchez-Jacob, Raquel Zubizarreta, Xavier Castells, the Cumulative False Positive Risk Group

**Affiliations:** 1 Department of Epidemiology and Evaluation, IMIM (Hospital del Mar Medical Research Institute), Barcelona, Spain; 2 Network for Research into Healthcare in Chronic Diseases (REDISECC), Madrid, Spain; 3 Basic Medical Sciences Department, Biomedical Research Institut of Lleida (IRBLLEIDA)-University of Lleida, Lleida, Spain; 4 Universitat Autònoma de Barcelona, Barcelona, Spain; 5 Navarra Breast Cancer Screening Programme, Public Health Institute, Pamplona, Navarra, Spain; 6 Oficina Tècnica de Cribratge, Corporació Sanitaria Parc Taulí-Institut Universitari Parc Taulí-UAB, Sabadell, Barcelona, Spain; 7 La Rioja Breast Cancer Screening Programme, Fundacion Rioja Salud, Logroño, La Rioja, Spain; 8 Dirección General de Programas Asistenciales, Consejería de Sanidad, Servicio Canario de Salud, Tenerife, Santa Cruz de Tenerife, Spain; 9 Foundation Society for Cancer Research and Prevention, Pere Virgili Health Research Institute, Reus, Tarragona, Spain; 10 Program & Analysis Unit, Health Office, Oviedo, Principado de Asturias, Spain; 11 General Directorate Public Health & Centre for Public Health Research, Valencia, Comunidad Valenciana, Spain; 12 Servicio de Promoción de la Salud y Programas Preventivos, Consejería de Sanidad, Valladolid, Castilla y León, Spain; 13 Galician Breast Cancer Screening Programme, Public Health & Planning Directorate, Health Office, Santiago de Compostela, La Coruña, Spain; Kyushu University, Japan

## Abstract

**Background:**

Breast cancer incidence has decreased in the last decade, while the incidence of ductal carcinoma in situ (DCIS) has increased substantially in the western world. The phenomenon has been attributed to the widespread adaption of screening mammography. The aim of the study was to evaluate the temporal trends in the rates of screen detected invasive cancers and DCIS, and to compare the observed trends with respect to hormone replacement therapy (HRT) use along the same study period.

**Methods:**

Retrospective cohort study of 1,564,080 women aged 45–69 years who underwent 4,705,681 screening mammograms from 1992 to 2006. Age-adjusted rates of screen detected invasive cancer, DCIS, and HRT use were calculated for first and subsequent screenings. Poisson regression was used to evaluate the existence of a change-point in trend, and to estimate the adjusted trends in screen detected invasive breast cancer and DCIS over the study period.

**Results:**

The rates of screen detected invasive cancer per 100.000 screened women were 394.0 at first screening, and 229.9 at subsequent screen. The rates of screen detected DCIS per 100.000 screened women were 66.8 at first screen and 43.9 at subsequent screens. No evidence of a change point in trend in the rates of DCIS and invasive cancers over the study period were found. Screen detected DCIS increased at a steady 2.5% per year (95% CI: 1.3; 3.8), while invasive cancers were stable.

**Conclusion:**

Despite the observed decrease in breast cancer incidence in the population, the rates of screen detected invasive cancer remained stable during the study period. The proportion of DCIS among screen detected breast malignancies increased from 13% to 17% throughout the study period. The rates of screen detected invasive cancer and DCIS were independent of the decreasing trend in HRT use observed among screened women after 2002.

## Introduction

Breast cancer is the most frequent tumour in women worldwide, and its incidence rates had risen steadily worldwide over these past decades [1]. However, since the early 2000's a downturn in its incidence rates have been reported in several developed countries [2]–[10]. The downturn has also been observed in Spain, more remarkably in women on the 45–69 age range [11], [12]. The phenomenon has been attributed to the widespread adaption of screening mammography once screening saturation was nearly achieved [11]–[13], as well as to the reduction in the use of hormone replacement therapy (HRT) among post menopausal women after the publication of the results of the Women's Health Initiative trial in 2002 [14]. The prevalence of HRT use in Spain has always been low compared to other countries [11], [15]–[17]. Furthermore, the decline in breast cancer incidence due to the reduction in HRT use has not been studied in Spain.

Different trends have been observed in the incidence of invasive cancer compared to ductal carcinoma in situ (DCIS). While the incidence of invasive cancer has declined in the last decade, the incidence of DCIS of the breast has increased in several countries [18]–[22]. DCIS have substantially increased in the proportion of breast malignancies detected. The increase has been attributed to the implementation of breast cancer screening [21], [23]. It is estimated that DCIS represents 20% of screen detected breast malignancies [21], [24].

The availability of individual level data from a cohort of screened women in Spain, followed during 15 years provides the opportunity to analyze the screen detected rates of invasive breast cancer and DCIS over time. We wanted to evaluate the temporal trends in the rates of screen detected invasive cancers and DCIS, and to study the temporal trends with respect to the HRT use along the same study period.

## Methods

### Ethics Statement

The study was approved by the Mar Teaching Hospital Research Ethics Committee. The data was analyzed anonymously and therefore no additional informed consent was required.

### Setting

The National Health System in Spain provides universal health coverage, including early detection of breast carcinoma. All women residing in Spain aged 50 to 69 years are actively invited to participate in population-based screening, with screening intervals every 2 years. However, some regions start inviting women at 45 years. Population-based breast cancer screening in Spain started in one region in 1990 and was implemented nationwide in 2005. Breast cancer screening in Spain follows the European Guidelines for Quality Assurance in Mammographic Screening [25] and its results meet the required standards [26]. Data from eight regions of Spain that perform population-based breast cancer screening were collected. The participating regions covered 44% of the Spanish target population for breast cancer screening in 2006. The participating women are provided with a unique personal identification number. Information about attendance, screening outcome, and diagnostic work-up was registered at an individual level in each screening region data base with the unique personal identification number.

### Study Population and Data Collection

Information was collected from 1,564,080 women aged 45 to 69 years of age who had undergone at least one biennial screening examination between 1992 and December 2006. The women underwent 4,705,681 screening examinations during the study period. Due to the small sample size, information on screening examinations performed in 1990 and 1991 was not used for the study.

At the time of each screening examination information is routinely collected related to the mammographic interpretation, whether or not the woman was recalled for additional evaluation to rule out or confirm malignancy, and the specific additional evaluations performed, if any. Additional evaluation for breast cancer assessment included additional mammography, magnetic resonance imaging, ultrasonography, fine-needle aspiration cytology, core-needle biopsy and open surgical biopsy. The diagnostic work-up for additional evaluation was carried out within a maximum of 2 months after screening. A definitive diagnosis of breast cancer was always histopathologically confirmed. Information on histopathology classification was routinely collected in the screening regions for screen detected cancers using the ICD-10 classification codes. A case was considered as screen detected if the diagnosis was made on the basis of a screening examination with subsequent diagnosis work-up procedures. Cases were classified as DCIS or invasive breast cancer.

In addition, information on HRT use was obtained through a questionnaire administered face-to face by a trained health professional at each screening visit immediately before the screening examination. Women were considered to be users of hormone replacement therapy at a screening examination if they reported to be current users or to have used hormone therapy in the sixth months previous to that visit.

### Statistical Analysis

Age-adjusted rates of screen detected invasive cancer and DCIS were calculated for first and subsequent screenings and 3-year period (1992–1994, 1995–1997, 1998–2000, 2001–2003, 2004–2006). The Age-adjusted rates of invasive breast cancer, DCIS, and HRT use among the screened women, were calculated for each calendar year. Age-specific incidence rates of invasive cancer and DCIS by 5 year age groups were computed standardized by first or subsequent screen. All age-standardizations were done using the direct method and the European standard population in 5-year age groups as reference.

Poisson regression analyses were used to estimate the trends in screen detected rates of invasive breast cancer and DCIS observed in the study population over the 15 year period. Calendar year, screening region, 5-year age groups and first/subsequent screen were used as explanatory variables. The estimated annual percentage change (APC) and 95% confidence intervals were obtained from the regression models. The APC was equal to 100 (e^m^ -1), where m is the coefficient of the variable of calendar year. Independent models were computed to evaluate the breast cancer trends of DCIS and invasive cancer separately, and to ascertain possible differences in the APC for first and subsequent screens.

In addition, changes in age- and region-adjusted detection rates of DCIS and invasive cancer over the study period were evaluated using transition change-point models [11], [27]. These models assume a Poisson distribution for the number of cases in each stratum and afford a statistical test for the existence of a change-point in the overall trend, and where this is the case, estimate the year in which the change-point is located and the APC before and after the change point. Overall significance level was set at P-value<0.05. Statistical analyses were performed using SAS 9.1 (SAS Institute, Cary, NC, USA) and R (R Foundation for Statistical Computing, Vienna, Austria).

## Results

Twenty nine percent of women were first screened at 45 to 49 years of age, and 30% at age 50 to 54 years (table 1). The crude number of screen detected cancers per 1,000 screening examinations increased with age, with and overall crude number of 2.73 per 1,000 screening examinations (table 1).

**Table 1:** Number of women screened, screening examinations, screen detected ductal carcinoma in situ (DCIS) and invasive breast cancers by 5-year age groups.

	Women screened^a^	Screening examinations^b^	Screen detected DCIS^c^	Screen detected invasive cancer^c^
Age	n	n	n (‰)	n (‰)
**45–49**	464,434	764,069	421 (0.55)	1649 (2.16)
**50–54**	477,084	1,219,228	571 (0.47)	2831 (2.32)
**55–59**	300,245	1,191,627	572 (0.48)	3076 (2.58)
**60–64**	260,264	1,084,986	554 (0.51)	3600 (3.32)
**65–69**	62,053	445,771	261 (0.59)	1695 (3.80)
**Overall**	1,564,080	4,705,681	2379 (0.51)	12,851 (2.73)

a Number of women with that given age at first screening examination.

b Number of screening examinations performed in women at that given age.

c ‰ calculated as number of cases per 1000 screening examinations.

A total of 16,309 screen detected cancers were diagnosed in the 1992–2006 period analyzed. Of these cancers 78.8% (n = 12,851) were invasive cancers, 14.6% (n = 2,379) were DCIS, and 6.6% (n = 1,079) were unknown. At first screen 6,845 cancers were detected (14.6% DCIS, 76.7% invasive and 8.8% unknown) and 9,464 at subsequent screens (14.6% DCIS, 80.3% invasive and 5.1% unknown). Mean (standard deviation) age at detection of DCIS was 56.7 (6.39) and for invasive cancers was 57.8 (6.23) (P value<0.001).

Overall age-adjusted screen detected cancer rates were higher at first screening compared with subsequent screens for both, DCIS and invasive cancer (table 2). The screen detected rates of DCIS increased by 3-year period for first and subsequent screens. The highest screen detected rate of invasive cancer was observed in the 1998–2000 period for first screen, and in the 1992–1994 period for subsequent screens (table 2).

**Table 2:** Age-adjusted incidence rates of screen detected ductal carcinoma in situ (DCIS) and invasive breast cancer (per 100,000 European standard population) by period and first or subsequent screen.

	First screen				Subsequent screen			
	DCIS		Invasive breast cancer		DCIS		Invasive breast cancer	
Period (3-years)	No. of cases	Rate	No. of cases	Rate	No. of cases	Rate	No. of cases	Rate
**1992–1994**	116	60.5	681	357.8	24	39.7	162	266.4
**1995–1997**	185	65.4	1,037	378.8	127	43.7	666	221.2
**1998–2000**	257	64.7	1,544	426.1	236	40.7	1,365	222.9
**2001–2003**	235	69.9	1,140	409.0	414	43.6	2,377	236.6
**2004–2006**	203	70.3	846	372.3	582	45.8	3,033	228.7
**Overall**	996	66.8	5,248	394.0	1,383	43.9	7,603	229.9

The overall age-specific rates of invasive cancer per 100,000 women-years increased with age. It was 215.8 for women aged 45–49 years, 232 at 50–54 years, 258 at 55–59 years, 332 at 60–64 years, and 380 at 65–69 years. The overall age-specific rates of DCIS per 100,000 women-years was 55 for women aged 45–49 years, 47 at 50–54 years, 48 at 55–59 years, 51 at 60–64 years, and 59 at 65–69 years.

**Figure 1:**
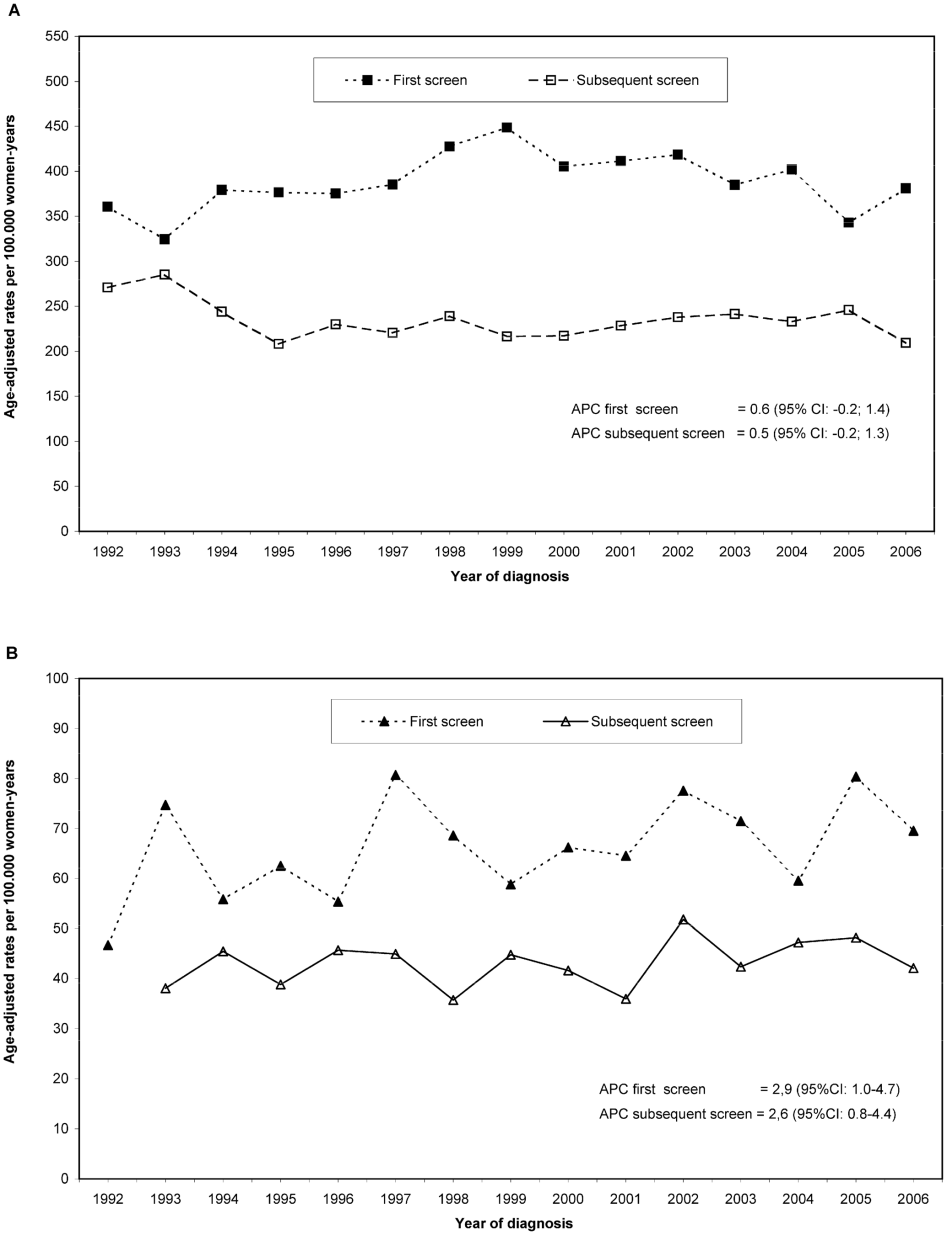
Age-adjusted rates of screen detected breast cancer for first and subsequent screens, in the 1992–2006 period. Rates are given per 100,000 women-years and are standardized using the European standard population in 5-year age groups as reference. The annual percentage change (APC) and its 95% confidence intervals (95%CI) were estimated from the Poisson regression model adjusted by age and screening region. **A**) Age-adjusted rates of invasive cancer for first and subsequent screens, and estimated APC and 95%CI. **B**) Age-adjusted rates of screen detected ductal carcinoma in situ (DCIS) for first and subsequent screens, and estimated APC and 95%CI.

After adjustment for age, screening region, and first or subsequent screen, the Poisson regression showed an absence of trend over the period studied for invasive cancers (p-value = 0.29), with a non-significant increase of 0.3% per year (APC 0.3, 95% CI: −0.2; 0.8), and a statistically significant increase of DCIS of 2.5% per year (APC 2.5, 95% CI: 1.3; 3.8). Figures 1a and 1b show the overall trends for first and subsequent screens for both, DCIS and invasive cancers. Fig. 1a shows that the incidence of screen detected invasive cancer was stable for first and subsequent screens with no significant trends over the period studied (p-value = 0.12 and 0.15 respectively for first and subsequent screens). As Fig. 1b depicts, the incidence of screen detected DCIS steadily increased along the study period for both, first and subsequent screens. The detection rates for DCIS increased by 2.9% per year for the first screen and 2.6% for the subsequent screens. There was no evidence of a change point in trend in the rates of DCIS and invasive cancers over the 17 year period studied (p-value for the existence of a change point = 0.3 for invasive cancer and p-value = 0.7 for DCIS).

Table 3 shows time trends for the rates of screen detected DCIS and invasive cancer by 5-year age groups over the study period. The P values refer to the evaluation of the existence of a change-point in the overall trend. Estimates for the APC and 95% CI were obtained from the Poisson regression model for each 5-year age group, adjusted for screening region and first or subsequent screen. There was no evidence of a change point in trend among any of the 5-year age groups, for neither invasive cancers nor DCIS. No significant APC was found for any 5-year age group over the study period for screen detected invasive cancers. The APC of screen detected DCIS showed a significant increase over the study period for the 45–49, 50–54, and 55–59 years age groups (table 3).

**Table 3:** Trends in rates of screen detected ductal carcinoma in situ (DCIS) and invasive breast cancer in the 1992–2006 period by 5-year age groups.

	DCIS			Invasive breast cancer		
	Change-point p-value^a^	Annual percentage change^b^		Change-point p-value^a^	Annual percentage change^b^	
Age		Overall	95% CI		Overall	95% CI
**45–49**	0.48	3.9	1.2; 6.5^c^	0.22	0.5	−0.8; 1.8
**50–54**	1.00	3.0	0.4; 5.6^c^	0.28	−0.3	−1.5; 0.8
**55–59**	1.00	2.8	0.1; 5.6^c^	0.25	1.1	−0.1; 2.3
**60–64**	1.00	0.8	−1.9; 3.6	0.99	0.6	−0.4; 1.7
**65–69**	1.00	1.7	−2.2; 5.7	1.00	0.6	−0.9; 2.2
**Overall**	0.65	2.5	1.3; 3.8^c^	0.29	0.3	−0.2; 0.8

a P-value for the existence of a change point in trend obtained from the Poisson transition change-point model adjusted by screening region. Analyses performed for each 5-year age group and for the overall.

b Annual percentage change and 95% confidence intervals (95%CI) obtained from the Poisson regression model adjusted by screening region and participation status (first or subsequent screen). Analyses performed for each 5-year age group and for the overall.

c Significant trend at the 95% CI.

**Figure 2:**
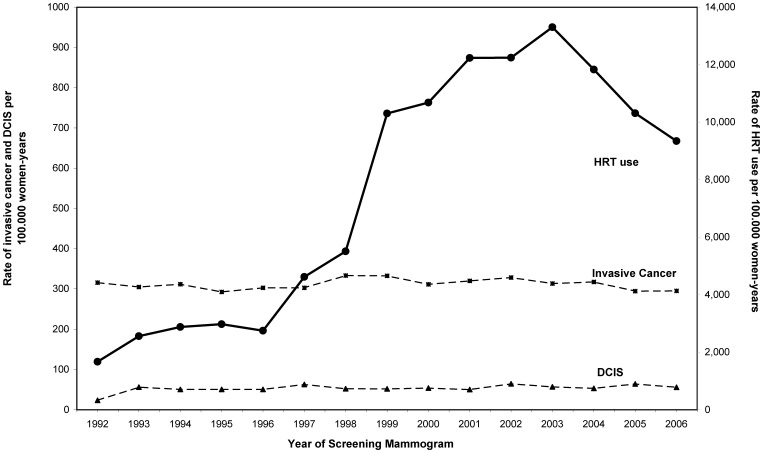
Rates of screen detected invasive cancer, screen detected ductal carcinoma in situ (DCIS), and hormone replacement therapy (HRT) use among screened women, in the 1992–2006 period. Rates are given per 100,000 women-years and are standardized to the age and first or subsequent screen using the European standard population in 5-year age groups as reference.

We presented data on HRT use by the screened women in our data set, obtained from the administered questionnaire at the time of screening examination. Information on HRT use was available in 69.3% of screening examinations. The percentage of missing information on HRT use was stable in the study period. An increase in the prevalence of HRT use was observed from 1992 up to 2003. HRT use from 1992 to 1996 was relatively low with a prevalence of 2,749 HRT users per 100,000 women-years in 1996 (fig. 2). A large increase in HRT use was observed from 1997 to 2003 when the prevalence level peaked (13,303 per 100,000 women-years). A decrease was observed after 2003, with a prevalence of HRT users of 9,344 per 100,000 women-years in 2006. A stable incidence of screen detected invasive cancer in the study period is shown in figure 2, independently of the HRT use among screened women. Similarly, the steady increase of 2.5% per year in the incidence of screen detected DCIS showed to be independent of the HRT use among the screened women.

## Discussion

Our results showed a steady increase of screen detected DCIS in Spain in the 1992–2006 period studied. The steady increase was observed for first and subsequent screens, and it was more markedly observed in screened women in the younger age groups. Despite the observed downturn in the population incidence of invasive cancer in women on the 45–69 age range the incidence of screen detected invasive cancers showed an absence of trend in the study period. The observed rates of screen detected DCIS and invasive cancer showed to be independent of HRT use among screened women.

The absence of trend in screen detected invasive cancers is in accordance with a previous study by Nederend et al. that reported an absence of trend in the rates of screen detected advanced cancers during a 12 year period [28]. With respect to DCIS, previous studies have shown an increase in the detection rates of DCIS. Van Steenbergen et al. found a ten-fold increase in the detection rate of DCIS between 1991 and 2000 in southern Netherlands, and a two-fold increase was found by Barchielli et al. in Italy [18], [22]. The widespread adaption of screening mammography has often been used to explain the increase in the incidence of DCIS in the general population found in several studies [18], [21], [22], [29], [30]. However, our study is targeted exclusively to screening participants and our findings should be interpreted in the screening setting. A reason for the increase in screen detected DCIS could be the changes in the techniques and interpretation of screening mammograms over time, as well as the changes in the pathological classification of pre-malignant breast lesions. Population-based screening in Europe follows the recommendations of the European guidelines [25], but programs have progressively improved their quality indicators and efficiency over the years. On the other hand, the introduction of digital mammography has increased the sensitivity of screening mammography, more markedly in the detection of DCIS [31]–[34]. However, less than 1.5% of screening test were performed with digital mammography in this study.

The steady increase in screen detected DCIS over the study period while the screen detected invasive cancers remained stable has caused that DCIS have substantially increased in the proportion of breast malignancies detected in screening mammography. The proportion has increased from 13% in 1994 to 17% in 2006. The observed proportion of screen detected DCIS among all malignancies observed in the last part of the period (17%) was similar to what has been reported (18%) in other European countries [21].

The rates of screen detected invasive cancer by 5-years age groups showed no trend after adjustment for screening region and first or subsequent screen. The absence of trend in the 5-years age groups reinforced the idea of a steady, stable detection rate of invasive cancers along the study period. On the other hand, the rates of DCIS by 5-years age groups showed a statistically significant increase for the 45–49, 50–54, and 55–59 years age groups. The estimated increase in the rates of DCIS in the three youngest age groups showed a decreasing gradient with age that ranged from 3.9% in the 45–49 years age group to 2.8% in the 55–59 years age group, and was not statistically significant for the 60–64, and 65–69 years age groups. Previous studies have also shown a highest proportion of DCIS among younger women [35].

The observed rates of screen detected DCIS and invasive cancer appeared to be independent of HRT use among screened women. An absence on change points in the overall trends was observed in all the analyses performed: screen detected DCIS and invasive breast cancer, first and subsequent screens, and 5-years age groups. If HRT use has had an effect in the screen detected rates of DCIS or invasive cancer we would expect to find a change point in the overall trends. The change in trend would be strongly expected after the year 2002 when the Women's Health Initiative trial was published [14], causing a reduction in the use of HRT among post menopausal women [17]. Figure 2 shows a reduction in the use of HRT starting in 2002, while the screen detected rates of DCIS and invasive cancer remain steady over the study period. Nevertheless, the time lag between the observed decreasing trend in HRT use and its impact in breast cancer incidence may be long. A reduction in screen detected breast cancer incidence may be observed in a longer term outside the end of our study period in 2006. However, we studied a four year offset from the reduction in the use of HRT in 2002 to the end of the study period in 2006. Several developed countries have reported data on population breast cancer incidence associated with a decrease in the use of HRT in shorter study periods, ranging from 2 to 5 years of offset [4]–[10]. On the other hand, longer duration of HRT use is known to increase women's breast cancer risk. However, an increased breast cancer risk is consistent for all estrogen plus progestin HRT users. The increased risk remains5-years or more after stop of HRT use [36]. In our study women were considered to be HRT users if they reported to be current users or to have used hormone therapy in the sixth months previous to the screening examination. The definition used ensures that HRT users had been users in a recent period (<6 months) avoiding misclassification of past users as current users.

We found that first and subsequent screens had similar trend patterns for both, invasive cancers and DCIS. By presenting the data for first and subsequent screens separately we avoided a potential confounding factor when analyzing long-term data for screen detected cancers. Higher screen detection rates were observed at first screens compared to subsequent screens, which was expected. However, the proportion of first and subsequent screens changes over time, with more first screenings performed as screening programmes are implemented during the study period, and in younger women who are first time invited. Not taking into consideration the participation when studying incidence trends in mammography screening may cause empirical estimators to be biased and confounded.

The widespread adaption of screening mammography once screening saturation was nearly achieve has been used to justify the observed downturn in the population incidence rates of invasive breast cancer reported since the early 2000 in women on the 45–69 age range [11], [12]. During the 1990s screening programs were implemented in the corresponding populations, and screening mammography was widespread adapted. Most programs achieved full coverage of the target populations during the late 1990s and early 2000's [12], [26], [37]. The steady, stable detection rate of invasive cancers along the study period found in this study does not support the downturn in the incidence rates of invasive breast cancer observed in the population since the early 2000 [4], [5], [7], [8], [10]. On the other hand, our findings could help to explain the increase in the population incidence of DCIS found in several studies [18]–[22]. The proportion of women in the population undergoing routine screening mammography will influence population-based estimates of breast cancer incidence [6]. The observed steady increase of DCIS in the proportion of screen detected breast malignancies from 13% to 17% is expected to influence the population incidence of DCIS. Previous studies have reported that over 67% of Spanish women in the 45–69 age range perform screening mammography in a publicly founded screening programme [26].

If the natural progression of invasive breast cancer is via DCIS, the detection of DCIS would help to prevent the development of breast carcinomas and consequently reduce breast cancer mortality [38]. However, the increasing number of screen detected DCIS, while the number of invasive cancers remains stable may present a clinical challenge if it implies an increase in the number of women overdiagnosed and overtreated [39].

Some limitations must be considered when interpreting our findings. Firstly, we did not have individual level data on non-participating women in the target population as we received anonymized data of screened women only from the participating regions. The attendance rate among invited women is reported to be 67% [26], and the re-attendance rate among participating women to be 91% [26]. The reported attendance and re-attendance rates are not dissimilar to other well established population-based screening programs in Europe [40]. It would have been desirable to have information on breast cancer risk factors among non-participating women. A previous study on usage of screening mammography previous to initiating a population-based breast cancer screening program in Spain showed that utilization of mammography was higher among younger women, women who had a higher education level, a family history of breast cancer, personal history of benign breast lesion, or had previous visits to a physician [41]. In addition, a substantial proportion of women in the 45–69 age range undergo opportunistic screening outside a screening programme [42]. Thus, the interpretations of the results in this study are related to detection in population-based screening, and its implication in the general population incidence should be carefully reviewed. However, a not dissimilar trend in screen detected DCIS and invasive cancer would be expected over time for population-based and opportunistic screening, as the changes in the interpretation of screening mammograms have occurred simultaneously. Besides, 6.6% of screen detected cancers in our study could not be classified as DCIS or invasive breast cancer because the histology classification was not available. The proportion of unknown histology of screen detected cancers decreased over time, as the screening programmes' databases achieved completeness and the established quality indicators were met. There were 9.5% unknown histology cancers cases in 1992, 6.2% in 1999, and 3.3% in 2006. To check whether the reduction in unknown histology cancer cases could have an effect in the observed increase in screen detected DCIS we performed a sensitivity analysis excluding the two screening regions with a highest proportion of unknown histology cancer cases at the beginning of the study period. No significant differences were observed compared to the analysis including all regions, therefore all cases were included in the analysis.

### Conclusions

We studied the trends in screen detected DCIS and invasive breast cancer over a 15 year period, and found that the studied rates were independent of HRT use among screened women. Despite the observed downturn in the population incidence of invasive cancers, the screen detected rates of invasive cancers remained steady, stable over the study period, while the screen detected rates of DCIS steadily increased, causing an increase of DCIS in the proportion of screen detected breast malignancies. The increasing trend of screen detected DCIS was associated to younger ages, particularly women aged 45–60 years. The study provides substantial information to improve the knowledge about the impact of screening programmes over time. These results are particularly useful when the benefits and harms of screening mammography are evaluated in the long-term.
